# An Introduction to Practical Pharmacy

**Published:** 1856-07

**Authors:** 


ART. VIII.—*An Introduction to Practical Pharmacy:* designed as a Text Book for the Student, and as a Guide to the Physician and Pharmaceutist. With many formulas and prescriptions. By EDWARD PARRISH, Graduate in Pharmacy, etc., etc. Philadelphia: BLANCHARD & LEA. 1856.

Mr. Parrish has done an essential service both to pharmaciens and physicians in the production of this book. We hardly know to whom it will be most useful, the prescription druggist, the prescribing physician, or the country physician who is obliged to be in many cases his own pharmaceutist, and needs a more accurate knowledge of practical pharmacy than the city practitioner.

The preliminary chapters are devoted to the furniture necessary to the dispensing shop, to weights, measures and specific gravity, and to the pharmacopœia. Then we have Galenical Pharmacy, the Pharmacy of Plants, their products, &c., the inorganic pharmaceutical preparations, several chapters on Extemporaneous Pharmacy and on the art of dispensing medicines, etc. The chapter on the writing of prescriptions is particularly useful and sensible, while the tables given f r apportioning quantities for select and combining medicines, &c., will all commend themselves to the practitioner, either in town or country.

The book is a handsome octavo of 550 pages, and will form a really valuable addition to any library, especially to that of the dispensing druggist or the physician who puts up his own medicines.

